# Multifocal Benign Metastasizing Pleomorphic Adenoma Presenting as a Lymphoma: An Atypical Clinical Picture Demystified by an Unusual *PLAG1* Gene Rearrangement Detected on RNA Next Generation Sequencing

**DOI:** 10.1002/dc.70098

**Published:** 2026-02-18

**Authors:** Poorva Singh, Spencer M. Erickson, Kathryn Baxstrom, Paari Murugan

**Affiliations:** ^1^ Department of Laboratory Medicine and Pathology, Medical School University of Minnesota Minneapolis Minnesota USA; ^2^ Internal Medicine Residency Program, Department of Medicine, Medical School University of Minnesota Minneapolis Minnesota USA; ^3^ HealthPartners Cancer Center at Regions Hospital Saint Paul Minnesota USA

**Keywords:** benign metastasizing, cytomorphology, NGS, *PLAG1*, pleomorphic adenoma

## Abstract

A 57‐year‐old female presented to urgent care with exertional dyspnea, back pain, and several months of night sweats. Imaging showed an anterior mediastinal mass with concurrent hepatic and vertebral lesions, raising suspicion for a hematolymphoid malignancy. Fine needle aspiration (FNA) and needle core biopsies were obtained from the liver lesion, with morphologic features compatible with pleomorphic adenoma (PA) without evidence of malignant or high‐grade cytologic features, adding to the complexity of interpretation. Revisiting her history revealed a diagnosis of a benign salivary gland tumor in the left parotid, which was surgically removed almost 40 years ago. This piece of clinical information put the morphology into perspective and subsequent molecular testing detected an unusual *PLAG1* gene rearrangement, confirming the diagnosis of a benign metastasizing PA in this unique case.

## 1 Introduction

Pleomorphic adenoma (PA) is the most common benign tumor of the salivary glands. In a small proportion of cases, it may undergo malignant transformation and, rarely, has been known to metastasize to distant sites without undergoing malignant transformation [1]. This case highlights a diagnostic challenge wherein a PA presented with a benign‐appearing multiorgan metastasizing occurrence decades after its initial presentation, clinically mimicking a clonal hematolymphoid process. It emphasizes the value of molecular analysis as an adjunct to morphology, especially in challenging scenarios.

## 2 Case Report

**FIGURE 1:**
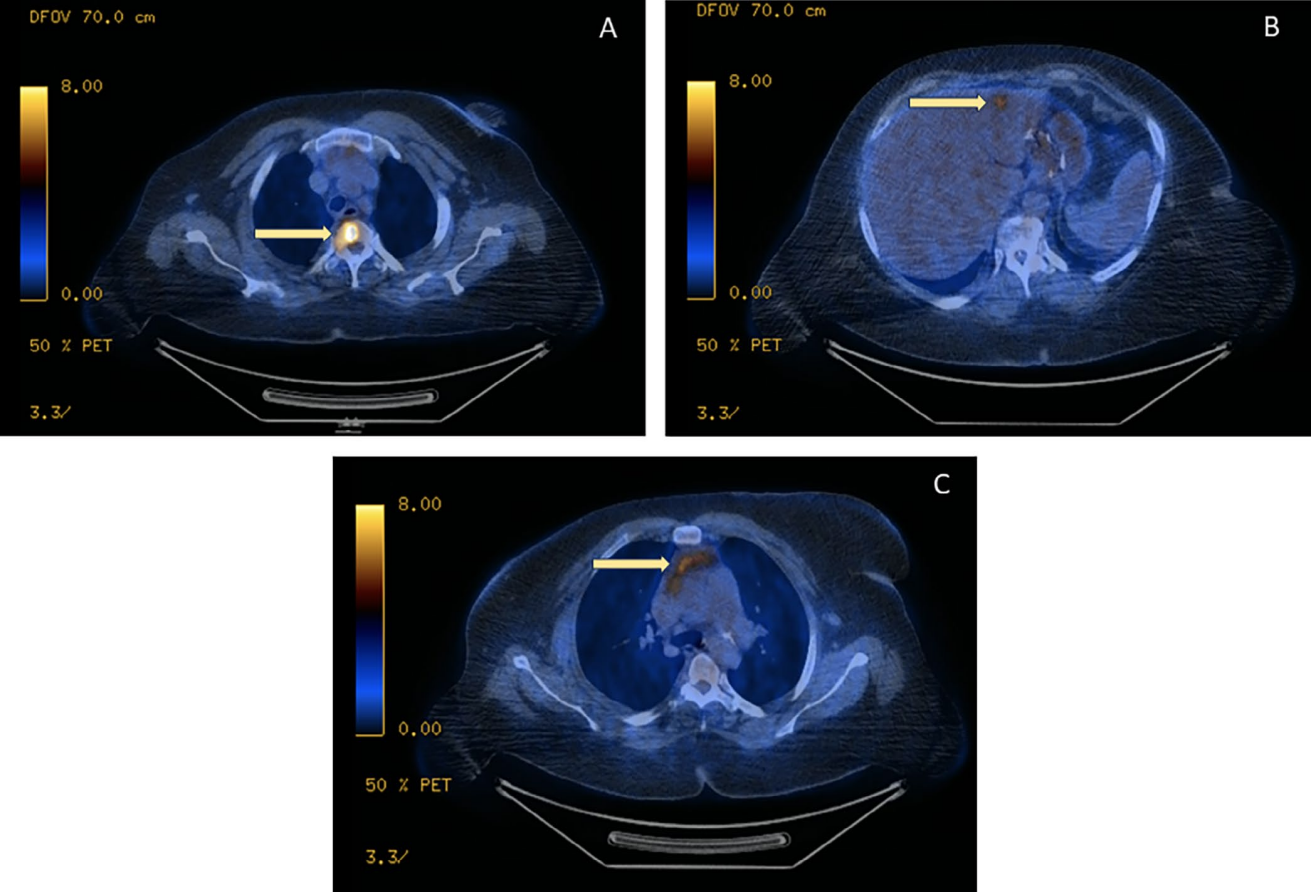
(A‐C). PET‐CT scan demonstrating FDG‐avid lesions at the T2 vertebra (SUV max 10.2) (A), left hepatic lobe (2.7 x 2 x 2.8 cm, SUV max 5.2) (B), and anterior mediastinum (3.6 x 1.5 cm, SUV max 3.1) (C), all highlighted by bold yellow arrows.

A 55‐year‐old female was referred to the oncology clinic after a recent hospitalization for dyspnea on exertion. She reported several months of drenching night sweats, focal upper chest pain, and chronic shortness of breath but denied hemoptysis or significant weight loss. Her medical history was remarkable for hypertension, polysubstance use, chronic back pain, obesity, iron deficiency anemia, bronchial asthma, and a 40 pack‐year smoking history. The physical exam was negative for lymphadenopathy, lower extremity edema, hepatosplenomegaly, or any findings of pulmonary compromise. She was, however, found to have mild diffuse enlargement of her thyroid. Given the high clinical suspicion for a hematolymphoid malignancy, a Positron Emission Tomography‐Computed Tomography (PET‐CT) scan was performed. Imaging revealed clinically significant fluorodeoxyglucose (FDG)‐avid lesions in the anterior mediastinum, the T2 vertebral body, and the left hepatic lobe (Figure 1 A‐C). During her hospital stay, she developed thyrotoxicosis and was subsequently diagnosed with Graves' disease; this was successfully managed with methimazole and propranolol.

**FIGURE 2:**
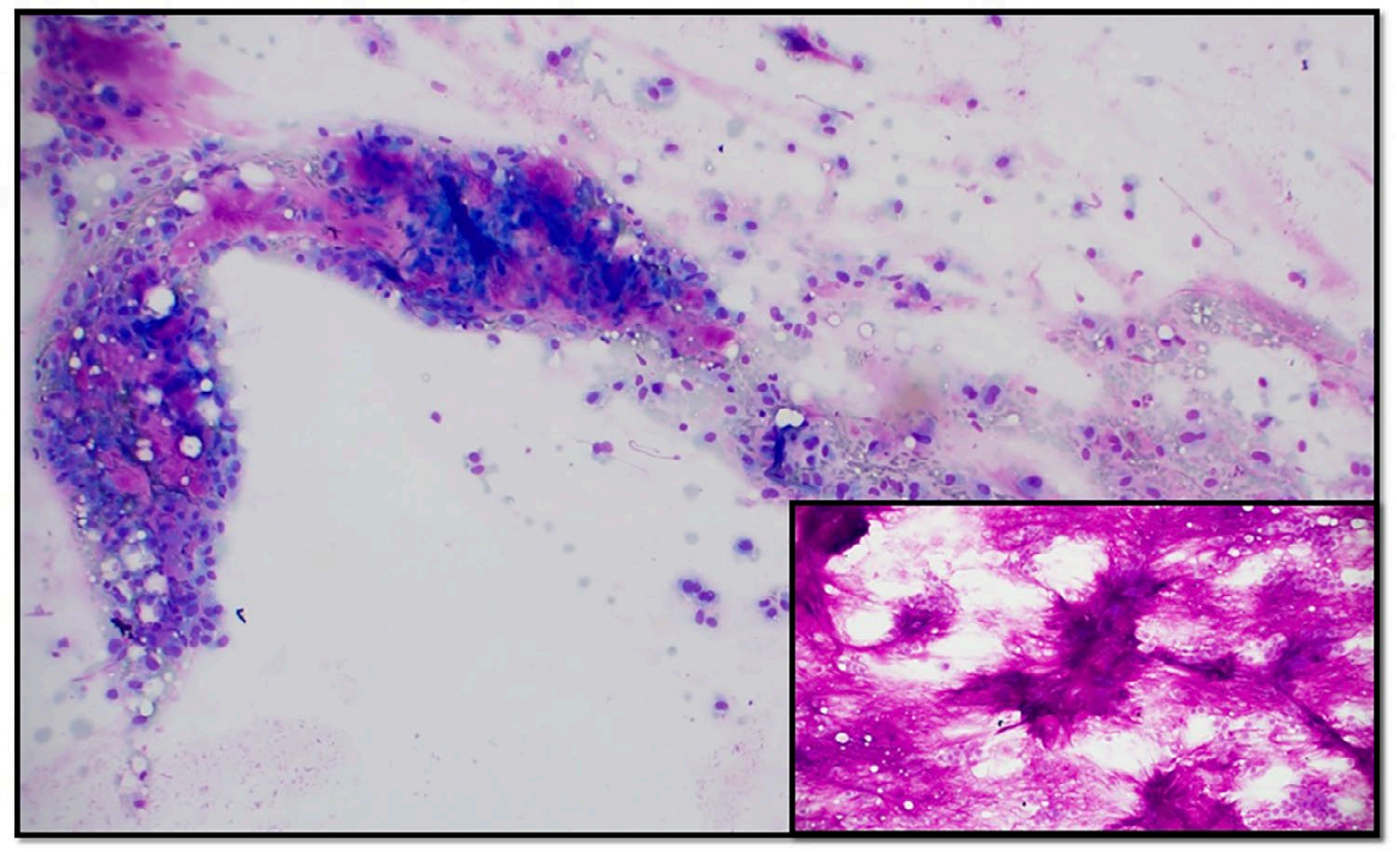
Cytology smear depicting a magenta fibrillary matrix intimately associated with bland epithelial cells and myoepithelial cells in the background, the characteristic cytomorphology of a benign salivary gland neoplasm, most notably a pleomorphic adenoma (Diff Quik, 100×), with the inset showing abundant fibrillary metachromatic matrix supporting benignity (Diff Quik, 200×).

Tissue sampling was deemed imperative to define the underlying pathology. A transcutaneous imaging‐guided fine needle aspiration (FNA) and core biopsy of the 2.8 cm liver lesion were obtained. Cytologic smears showed groups of bland‐appearing epithelial cells intimately associated with abundant metachromatic magenta fibrillary stroma (Figure 2) with bare bipolar oval nuclei scattered in the background. The epithelial cells were small‐ to medium‐sized with moderate amount of pale blue agranular cytoplasm, round to oval nuclei having uniform chromatin and inconspicuous nucleoli. There was no evidence of marked cellular atypia, brisk mitotic activity or dirty necrosis. This cytomorphology was highly suggestive of a benign salivary gland neoplasm, and most likely a PA, given the intimate association of epithelial cells with myxofibrillary metachromatic matrix.

**FIGURE 3:**
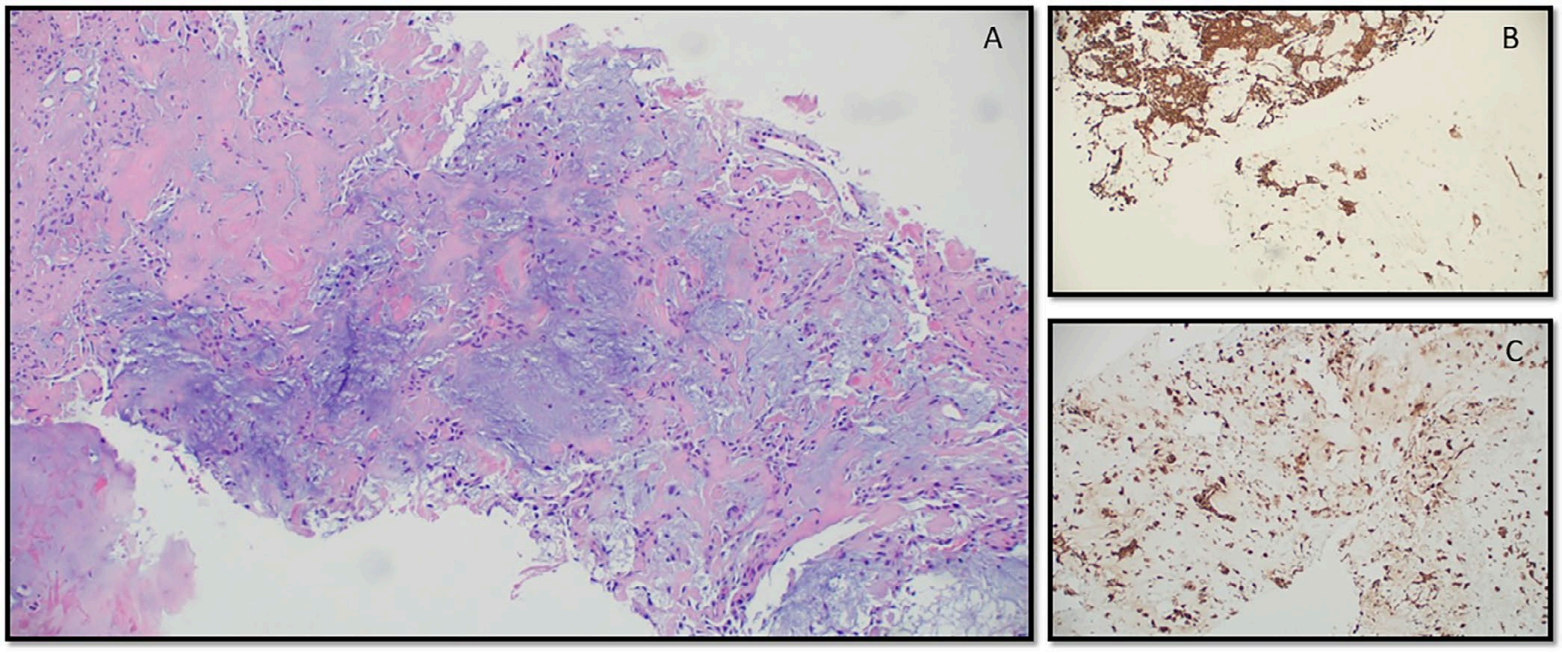
(A‐C). Core biopsy demonstrating abundant chondromyxoid stroma with the cellular component consisting of interspersed small, bland appearing round to oval cells (HE, 100×) (A); Pancytokeratin highlighting the dual population of cells—the epithelial component showing brown cytoplasmic staining and the myoepithelial component seen as bluish nuclei, lacking any positivity (IHC, 100×) (B); Myoepithelial component highlighted by S‐100, with positivity in single cells and small groups of cells embedded in the stromal component (IHC, 100×) (C), all findings consistent with a pleomorphic adenoma.

The concurrent core biopsy exhibited a biphasic morphology—a bland epithelial component closely associated with a stromal component best characterized as “chondromyxoid” with areas of hyalinization (Figure 3A). This stroma contained small, bland appearing round to oval nuclei. Again, there was a lack of high‐grade nuclear features, brisk mitoses, or necrosis. Immunohistochemical (IHC) stains were performed—pancytokeratin was strongly positive in the epithelial cells (Figure 3B) while S‐100 was diffusely positive in the stromal cells, highlighting a dual population of epithelial and myoepithelial cells (Figure 3C). GFAP, calponin, and SMA also showed patchy reactivity in the stromal cells, characteristic of myoepithelial differentiation in salivary gland tumors. CD31, p40, and ERG were negative in both components.

**FIGURE 4:**
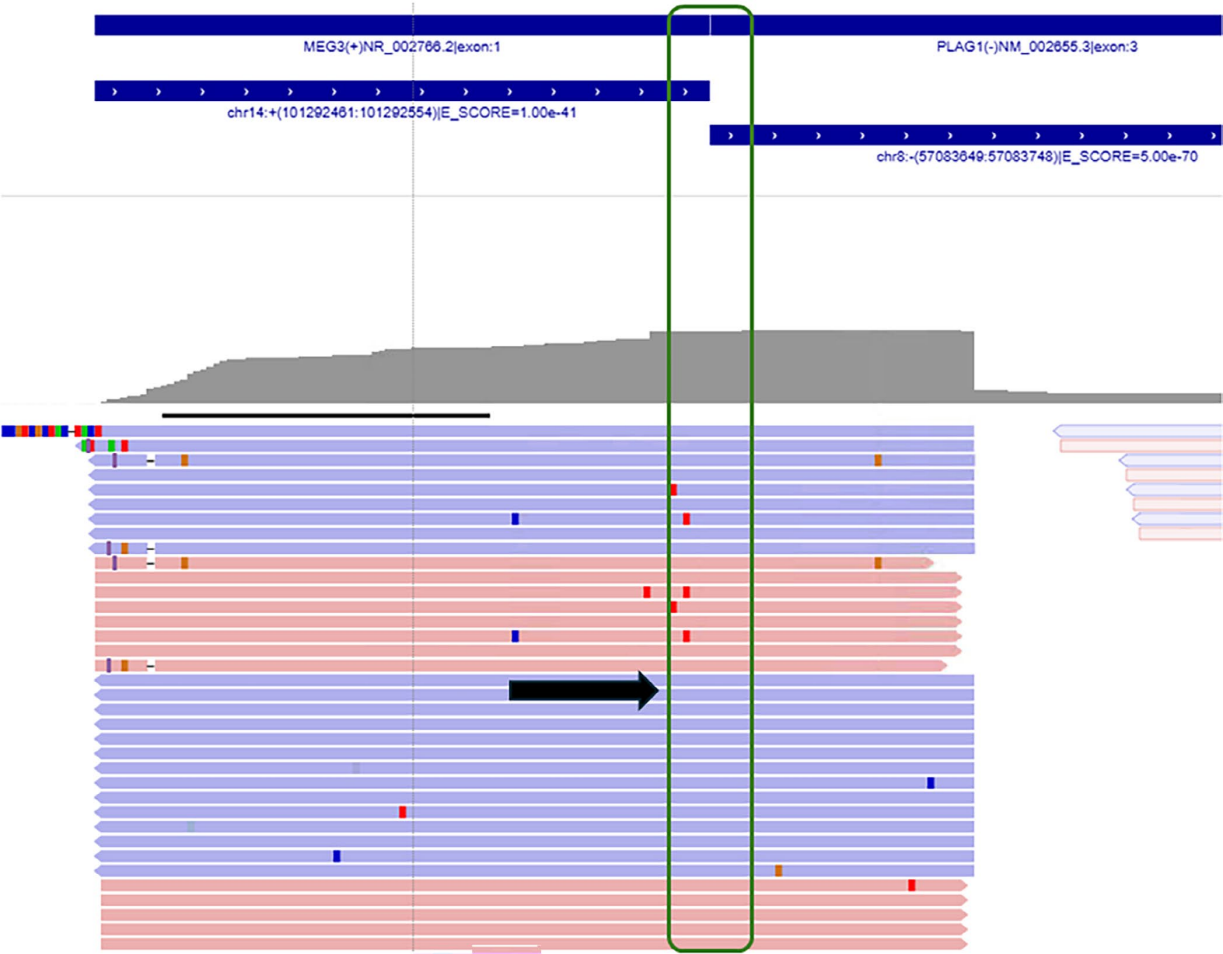
RNA sequencing data show numerous reads spanning across the breakpoint between exon 1 of *MEG3* on the 5′ end and exon 3 of *PLAG1* on the 3′ end (thick arrow and box, Integrated Genomics Viewer).

The morphologic findings were most compatible with a PA. Despite the convincing morphology, molecular testing was considered the appropriate next step, given the ominous clinical picture of multiple FDG‐avid lesions and night sweats, with a striking lack of any concurrent or past salivary gland pathology. A targeted RNA‐based Next Generation Sequencing (NGS) assay was performed to look for rearrangements involving *PLAG1* or *HMGA2* genes, which are known recurring genetic alteration(s) in PA and other benign salivary neoplasms [2]. While the care team was awaiting NGS results, a more thorough history was elicited from the patient during follow‐up. She did recall undergoing a left parotidectomy approximately 40 years ago for a “benign” salivary gland tumor, the records of which were unavailable for review. As expected, her RNA NGS came back positive for *MEG3::PLAG1* gene fusion (Figure 4), a novel genetic rearrangement [3], previously undocumented in benign metastasizing PA.

**FIGURE 5:**
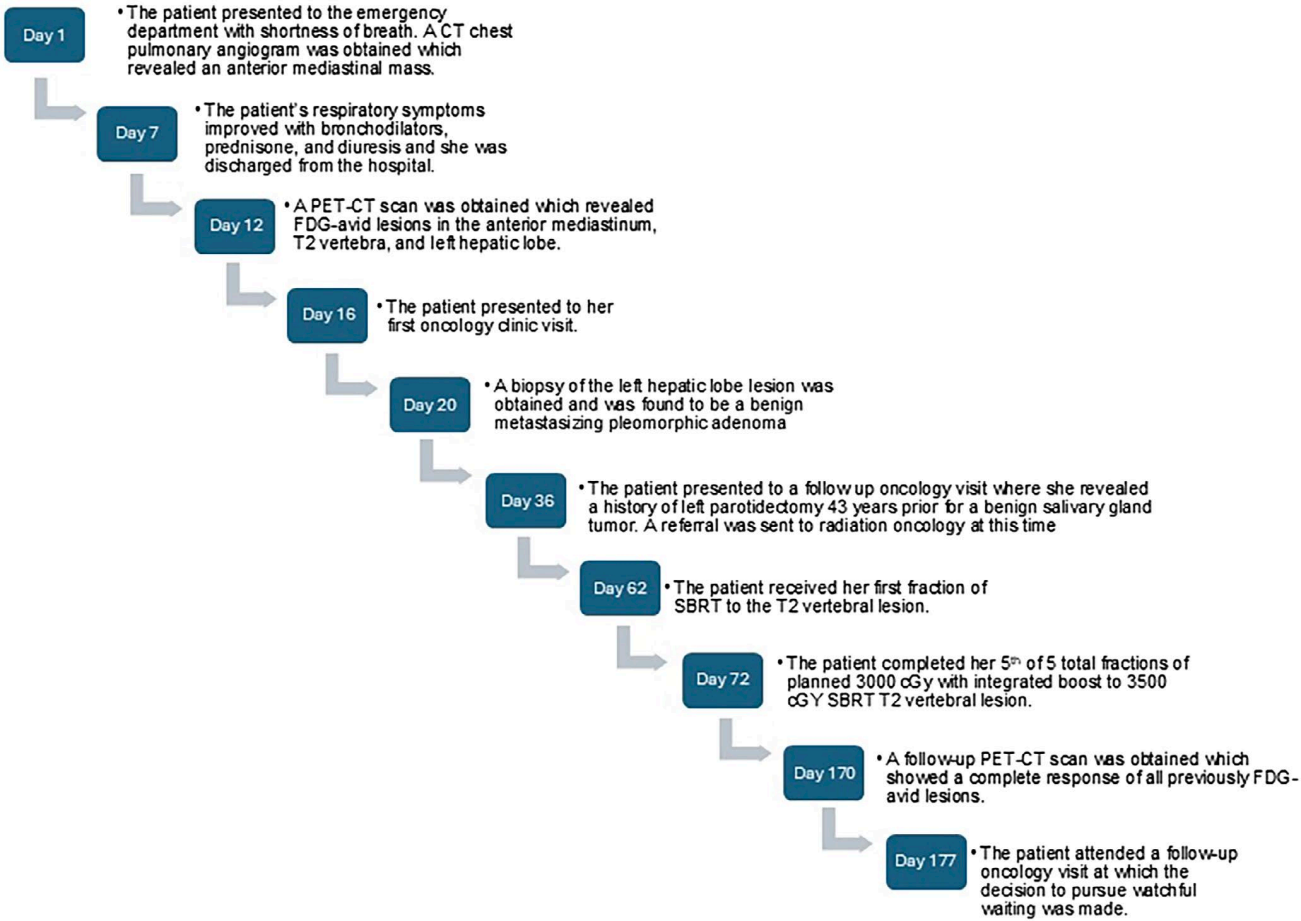
Timeline of the patient's clinical course.

After a shared decision‐making process, tissue sampling of vertebral and mediastinal lesions was not pursued, and the patient elected to undergo palliative radiotherapy (RT) to the vertebral lesion. A PET‐CT scan 3 months later revealed a complete response at all three hypermetabolic sites. She elected to pursue a watchful waiting approach and continues to be observed for evidence of recurrence (Figure 5).

## 3 Discussion

PA has been described in organs other than the salivary gland without undergoing histologic signs of malignant transformation [1], thus earning the name, “benign metastasizing pleomorphic adenoma (BMPA).” A search of medical literature from 1980 to 2025, returned approximately 75 published cases of BMPA, and most had a prior history of excision of PA in a major or minor salivary gland and occasionally, had synchronous involvement of other sites [2, 4]. The average age at diagnosis was 49 years and the average time to metastasize was 17 years [1, 4, 5, 6] However, there is ongoing debate regarding whether this entity is a benign neoplasm that spreads to extra‐salivary sites via hematogenous or lymphatic route after surgical manipulation or via aspiration, as some authors have suggested that it likely represents a continuum of carcinoma ex‐PA (malignant transformation within a biopsy‐proven PA), and should instead be considered a truly malignant process [1, 7]. Interestingly, the clinical picture of many of these rare occurrences has been consistent with that of a low‐grade malignant process, with most patients (> 75%) experiencing disease‐free periods after surgical excision and RT [1, 4], with a small number succumbing to metastatic disease.

Our present case reinforces the theory that BMPAs are indolent low‐grade neoplasms, but this theory needs to be confirmed on long‐term follow‐up. Our patient's multifocal disease most likely represented a late benign metastasizing presentation of the previously resected benign salivary gland tumor of the parotid gland, which was presumably a PA. Although not confirmed on tissue biopsy in accordance with the patient's wishes, it was clinically inferred that all three FDG‐avid discrete masses were part of the same neoplastic process. Bone and mediastinum have been previously described in published literature as usual sites of involvement by BMPA [8]. There have also been occasional published case reports of BMPAs presenting as multifocal lesions clinically manifesting decades after the initial presentation [7], supporting this theory.

BMPAs presenting as anterior mediastinal masses have been reported previously [4, 8]. However, our case was unique due to several reasons—first, the patient's alarming symptomatology of drenching night sweats and multiple FDG‐avid lesions which mimicked a malignant process; second, there was an initial lack of evidence of prior salivary gland pathology that was elicited much later in the clinical course after morphologic assessment had already happened; and thirdly, the morphologic diagnosis was confirmed by NGS testing which revealed a novel *MEG3::PLAG1* gene rearrangement. While the *PLAG1* gene rearrangement is highly sensitive and specific for PA [2, 3], only about 10BMPA cases have been confirmed to harbor *PLAG1* or *HMGA2* rearrangements with RNA sequencing [1, 2, 6, 9, 10].

## 4 Conclusion

This report highlights an unusual presentation and rearrangement in a benign metastasizing PA, emphasizing the combined value of robust cytomorphologic evaluation, immunohistochemical analysis, and molecular testing. This rare entity presented after a long interval of four decades from the initial diagnosis, also underscoring the importance of effective history taking in challenging clinical situations.

## Author Contributions

Poorva Singh – conceptualization (lead), data curation (supporting), writing – original draft (equal), writing – review and editing (lead). Spencer M. Erickson – writing – original draft (equal), data curation (lead), conceptualization (supporting). Kathryn Baxstrom – supervision (equal), conceptualization (supporting). Paari Murugan – supervision (equal), conceptualization (supporting), writing – original draft (supporting).

## Funding

The authors have nothing to report.

## Conflicts of Interest

S.M. Erickson was awarded $750 for presentation of parts of this case at the Minnesota Society of Clinical Oncology 2024 Spring Conference on April 24, 2024, in Minneapolis, MN. All other authors declare no relevant conflicts of interest.

## Data Availability

The data that support the findings of this study are available from the corresponding author upon reasonable request.
